# Repeat Kidney Biopsy in Lupus Nephritis: Diagnostic Yield and Clinical Utility in a Real-World Single-Centre Cohort

**DOI:** 10.3390/jcm15145515

**Published:** 2026-07-14

**Authors:** Marc Patricio-Liébana, Maria José Soler, Alejandra Gabaldón, Sheila Bermejo, Sara Núñez, Marina López, Abel Andrés Orelogio, Josefina Cortés-Hernández, Oriol Bestard, Irene Agraz

**Affiliations:** 1 Centro de Referencia CSUR para Patología Glomerular Compleja, Servei de Nefrologia, Vall d’Hebron Hospital Universitari, 08035 Barcelona, Spain; marc.patricio@vallhebron.cat (M.P.-L.);; 2 Vall d’Hebron Institut de Recerca, 08035 Barcelona, Spain; 3 Servei d’Anatomia Patològica, Vall d’Hebron Hospital Universitari, 08035 Barcelona, Spain; 4 Unitat de Lupus, Servei de Reumatologia, Vall d’Hebron Hospital Universitari, 08035 Barcelona, Spain

**Keywords:** systemic lupus erythematosus, lupus nephritis, kidney biopsy

## Abstract

**Background/Objectives:** Repeat kidney biopsy in lupus nephritis is increasingly used to resolve discrepancies between clinical, serological and histological findings, but its diagnostic yield in real-world practice remains incompletely defined. This study aimed to describe the indications, safety, histological transitions, clinically relevant findings, treatment changes and clinical course of patients with lupus nephritis undergoing repeat native kidney biopsy. **Methods:** We conducted a retrospective single-centre study including adults with biopsy-proven lupus nephritis attended between 2003 and 2025 who underwent at least one repeat native kidney biopsy. Analyses were performed at patient level, comparing the index and last biopsy, and at episode level, evaluating consecutive biopsy transitions. **Results:** Nineteen patients and 45 native kidney biopsies were included. The most frequent indication for repeat biopsy was serological and/or proteinuric worsening. Comparing the index and last biopsy, histological or diagnostic change was observed in 14/19 patients (73.7%), including diagnoses not compatible with active lupus nephritis in 3/19 (15.8%). Sequential analysis identified 26 transitions; 17/26 (65.4%) showed lupus nephritis class change, 4/26 (15.4%) incident membranous component and 3/26 (11.5%) a diagnosis that was not compatible with active lupus nephritis. Two biopsy-related hematomas occurred and three patients required kidney replacement therapy during follow-up. **Conclusions:** In this real-world cohort, repeat kidney biopsy provided clinically relevant and potentially actionable diagnostic information, particularly through sequential analysis of consecutive biopsies.

## 1. Introduction

Lupus nephritis (LN) remains one of the most prognostically relevant manifestations of systemic lupus erythematosus (SLE), as it is associated with progression to chronic kidney disease, need for kidney replacement therapy and increased morbidity and mortality [1,2,3,4,5,6]. Kidney biopsy remains the reference standard for classifying renal involvement, estimating the balance between inflammatory activity and chronic damage, and informing the intensity and mechanism of immunosuppressive treatment [1,3,7,8,9].

In recent years, the therapeutic management of lupus nephritis has evolved toward more stringent goals of proteinuria control, preservation of kidney function and maintenance of remission [1,2,8,10,11,12]. This evolution has been driven by the incorporation of new therapeutic options and by improved definitions of clinical response targets [13,14,15,16,17,18,19,20,21,22,23,24,25]. However, the correlation between conventional clinical biomarkers and renal histology remains limited [3,9,26,27,28,29]. Proteinuria, kidney function and immunological markers do not always reliably distinguish active inflammatory lesions, established structural damage or alternative diagnoses [3,9,26,27,28,29]. In this context, repeat kidney biopsy has gained increasing interest in different clinical scenarios, including suspected flare, suboptimal response and protocol biopsy in apparent clinical remission [26,27,28,29,30,31,32,33,34,35,36,37,38]. Several studies have shown that histological activity may persist despite clinical improvement and that residual activity may be associated with renal relapse, increasing chronicity and worse long-term kidney outcomes [27,28,29,30,31,32,33,39,40,41,42,43,44]. Repeat biopsies may also reveal histological class transitions, incident membranous component or, in some cases, findings not compatible with active lupus nephritis, with potential implications for intensifying, maintaining or reducing immunosuppressive therapy [26,30,31,34,35,36,37,38].

Nevertheless, much of the available evidence comes from protocolized cohorts, selected indications, or studies focused on specific prognostic endpoints, which may limit extrapolation to routine clinical practice [26,28,30,31,32,33,39,40,44]. In this setting, it is clinically relevant to describe what information repeat kidney biopsy provides in real-world care, in which scenarios it is performed and which types of diagnostic findings it identifies [26,34,35,36,37,38]. Therefore, the aim of this study was to describe the indications, safety, histological transitions, clinically relevant diagnostic findings, treatment changes and clinical course of a single-centre cohort of patients with lupus nephritis undergoing repeat native kidney biopsy in routine clinical practice. The added value of this study lies in assessing repeat kidney biopsy from a dual perspective: at patient level, by comparing the index biopsy with the last available biopsy, and at episode level, by evaluating histological transitions between consecutive biopsies. This approach may identify clinically relevant intermediate changes that could be missed by analyses restricted to the first and last biopsy [39,40,43,44].

## 2. Materials and Methods

This was a retrospective, observational, single-centre study of adult patients with systemic lupus erythematosus fulfilling 2019 EULAR/ACR criteria [45] and biopsy-proven lupus nephritis followed at Vall d’Hebron Hospital Universitari between July 2003 and July 2025. Eligible patients were required to have an index native kidney biopsy and at least one subsequent repeat native kidney biopsy. Non-diagnostic biopsies and biopsies with insufficient information to assign a histological diagnosis were excluded. Clinical, laboratory and histological data were retrospectively collected from electronic medical records. The following variables were recorded: demographic characteristics, number and dates of kidney biopsies, indication for each repeat biopsy, biopsy-related complications, need for kidney replacement therapy and death during follow-up.

At the time of the index biopsy, each repeat biopsy and the last available follow-up, the following clinical and laboratory parameters were collected when available: serum creatinine, estimated glomerular filtration rate, proteinuria expressed as urine protein-to-creatinine ratio (UPCR), hematuria, leukocyturia, antinuclear antibodies, anti-double-stranded DNA antibodies, and C3 and C4. Twenty-four-hour proteinuria values were reviewed when available but were not included in comparative analyses because they were not systematically obtained, and the number of serial determinations was limited. This approach was chosen to preserve analytical homogeneity. Extra-renal SLE manifestations at the time of the repeat biopsy were reviewed when available but were not uniformly captured across the study period; therefore, they were not included in formal comparative analyses.

The indication for repeat kidney biopsy was classified at episode level into four predefined categories based on the main clinical reason documented in the medical record: serological and/or proteinuric worsening, planned reduction or withdrawal of immunosuppression, refractory disease, and inclusion in a clinical trial. Given the retrospective design and the absence of a uniform protocol for repeat biopsy, categories were assigned according to the primary indication recorded by the treating team at the time of biopsy.

Histology was classified according to the International Society of Nephrology/Renal Pathology Society (ISN/RPS) classification reported in the pathology report issued at the time of each biopsy [7]. Proliferative forms were defined as class III, class IV and mixed class III + V or IV + V lesions [7]. No centralized retrospective pathological review was performed; therefore, the results reflect the histological interpretation available in real-world clinical practice. A diagnosis not compatible with active lupus nephritis was defined as any repeat biopsy whose pathology report did not correspond to an active class of lupus nephritis according to the ISN/RPS classification [7].

At episode level, transitions between consecutive biopsies were analyzed. Class change among biopsies classified as lupus nephritis was defined as any modification in ISN/RPS class between two consecutive biopsies with a diagnosis of active lupus nephritis, including changes between proliferative classes, between proliferative and non-proliferative active forms, or the appearance or disappearance of mixed forms. Incident membranous component was defined as the identification of class V lesions in a biopsy in which this component was not present in the immediately preceding biopsy. For descriptive purposes, a clinically relevant diagnostic finding was defined as the presence of at least one of the following elements in a given transition: class change among biopsies classified as lupus nephritis, incident membranous component, or a diagnosis not compatible with active lupus nephritis. A high-value diagnostic finding was defined as the concurrence of at least two of these criteria in the same transition.

SLEDAI and renal SLEDAI scores were not systematically recorded at the time of repeat biopsy. Although UPCR, urinary sediment variables, anti-dsDNA antibody titres and complement levels were collected when available, the retrospective design did not allow consistent temporal matching between repeat kidney biopsy and all clinical, urinary and serological parameters considered markers of lupus nephritis activity. The limited sample size and incomplete standardized capture of these variables therefore precluded formal association analyses with histological progression or specific ISN/RPS classes.

### Statistical Analysis

Continuous variables were summarized as median and interquartile range, and categorical variables as number and percentage. Paired comparisons of proteinuria between diagnosis and the second biopsy, the last biopsy and the last available clinical follow-up were performed using the Wilcoxon signed-rank test.

Given the retrospective design, small sample size and descriptive purpose of the study, no multivariable models were fitted. Analyses were performed using JASP version 0.19.3. A two-sided *p* value < 0.05 was considered statistically significant.

## 3. Results

### 3.1. Baseline Characteristics

Nineteen patients with lupus nephritis who underwent at least one repeat native kidney biopsy were included. Overall, 45 native kidney biopsies were analyzed. The median number of biopsies per patient was two, with a range from two to five biopsies. The cohort was predominantly female and showed a predominance of proliferative forms at index biopsy. Baseline characteristics of the cohort are shown in Table 1.

A total of 16 patients were women (84.2%). The median age at systemic lupus erythematosus diagnosis was 33.0 years (21.5–40.0), and the median age at lupus nephritis diagnosis was 35.0 years (24.0–41.0). The median follow-up time until the last biopsy was 60.2 months (50.4–114.5). Regarding histological class at index biopsy, class III was present in eight patients (42.1%), class IV in six patients (31.6%), class I in three patients (15.8%) and class III + V in two patients (10.5%). Median baseline serum creatinine was 0.81 mg/dL (0.62–1.44), median estimated glomerular filtration rate was 90 mL/min/1.73 m^2^ (53–90), and median baseline proteinuria was 912 mg/g (683.4–2000.5). No repeat biopsy included in the analytical cohort was reported as showing thrombotic microangiopathy or renal vasculitic lesions as a histopathological diagnosis.

### 3.2. Indications for Repeat Kidney Biopsy

The most frequent indication for repeat kidney biopsy was serological and/or proteinuric worsening, accounting for 13 episodes (50%). The second most frequent indication was planned reduction or withdrawal of immunosuppression, accounting for seven episodes (26.9%). Repeat biopsy was less frequently performed because of refractory disease or in the context of inclusion in a clinical trial, with three episodes each (11.5%). The distribution of indications is shown in Table 2.

### 3.3. Safety and Clinical Events During Follow-Up

Biopsy-related complications were recorded in 2 of 19 patients, both consisting of hematoma. Expressed per procedure, hematomas were documented in 2 of 45 biopsies. During follow-up, 3 of 19 patients required kidney replacement therapy. No deaths were documented.

### 3.4. Patient-Level Analysis: Index Versus Last Biopsy

When comparing the index biopsy with the last available biopsy, histological or diagnostic change was observed in 14 of 19 patients (73.7%). Lupus nephritis class change was observed in 11 of 19 patients (57.9%). In 3 of 19 patients (15.8%), the last biopsy showed a diagnosis not compatible with active lupus nephritis. The diagnoses recorded in this subgroup were fibrillary glomerulonephritis, glomerulosclerosis and optically normal biopsy. The appearance of a membranous component that was not present at the index biopsy was identified in 2 of 19 patients (10.5%). The diagnostic yield at patient level is summarized in Table 3.

### 3.5. Episode-Level Analysis: Consecutive Biopsy Transitions

Twenty-six transitions between consecutive biopsies were analyzed. Lupus nephritis class change was observed in 17 of 26 transitions (65.4%). The appearance of a new membranous component compared with the immediately preceding biopsy was documented in 4 of 26 transitions (15.4%). In 3 of 26 transitions (11.5%), repeat biopsy showed a diagnosis that was not compatible with active lupus nephritis.

When clinically relevant diagnostic finding was defined as the presence of at least one of the following elements—lupus nephritis class change, incident membranous component or diagnosis not compatible with active lupus nephritis—20 of 26 transitions (76.9%) showed at least one such finding. When a high-value diagnostic finding was defined as the concurrence of at least two of these criteria in the same transition, this occurred in 4 of 26 episodes (15.4%).

### 3.6. Incident Membranous Component During Follow-Up

The appearance of a new membranous component was identified in 2 of 19 patients when comparing the index biopsy with the last biopsy. However, in the sequential analysis of all transitions, incident membranous component was identified in 4 of 19 patients during follow-up. In two patients, a calcineurin inhibitor was initiated after repeat biopsy showed class V lesions.

### 3.7. Diagnostic Yield According to Clinical Indication

In descriptive analyses, diagnostic yield differed numerically according to the clinical indication for repeat biopsy; no formal between-group statistical comparison was performed because of the small number of episodes in each category. Episodes prompted by serological and/or proteinuric worsening constituted the most frequent group and showed at least one clinically relevant diagnostic finding in 9 of 13 transitions (69.2%). In this subgroup, lupus nephritis class change was observed in 7 of 13 episodes (53.8%), incident membranous component in 2 of 13 episodes (15.4%) and diagnosis not compatible with active lupus nephritis in 2 of 13 episodes (15.4%).

In repeat biopsies performed in the context of planned reduction or withdrawal of immunosuppression, at least one relevant diagnostic finding was identified in all analyzed episodes. Lupus nephritis class change was observed in six of seven episodes (85.7%), incident membranous component in one of seven episodes (14.3%) and diagnosis not compatible with active lupus nephritis in one of seven episodes (14.3%). Episodes performed because of refractory disease or inclusion in a clinical trial were less frequent but also showed clinically relevant findings in a substantial proportion of cases. In refractory disease, at least one relevant diagnostic finding was observed in two of three episodes. In episodes linked to clinical trial inclusion, at least one relevant diagnostic finding was observed in two of three episodes. The detailed distribution of findings according to indication is shown in Table 4.

### 3.8. Clinical Course and Treatment Changes After Repeat Kidney Biopsies

Proteinuria did not show significant differences between diagnosis and the second biopsy (*p* = 0.7086), nor between diagnosis and the last biopsy (*p* = 0.6794). In the comparison between diagnosis and the last available clinical follow-up, lower proteinuria values were observed at the final follow-up visit (*p* = 0.0032).

Treatment changes after repeat kidney biopsy are summarized in Table 5. A direct modification of immunosuppressive therapy was recorded after 16 of 26 repeat-biopsy episodes (61.5%). Immunosuppression was escalated or intensified in 10 episodes (38.5%), whereas immunosuppression was reduced or withdrawn in six episodes (23.1%). In six episodes (23.1%), no treatment reduction was performed, and the current therapeutic strategy was maintained. In two episodes (7.7%), repeat biopsy was followed by clinical trial inclusion, and in two additional episodes (7.7%) the documented consequence was a non-immunosuppressive clinical action, consisting of angiotensin-converting enzyme inhibitor initiation or kidney replacement therapy initiation. Thus, a clinical or therapeutic consequence was documented after 20 of 26 repeat-biopsy episodes (76.9%), while six episodes resulted in maintenance of the current treatment strategy without immediate therapeutic reduction.

When analyzed according to indication, all repeat biopsies performed for refractory disease led to immunosuppression escalation. Among repeat biopsies performed before planned immunosuppression reduction, treatment was reduced or withdrawn in two of seven episodes (28.6%), whereas no treatment reduction was performed in five of seven episodes (71.4%). In episodes prompted by serological and/or proteinuric worsening, repeat biopsy led to heterogeneous consequences, including immunosuppression escalation in 6 of 13 episodes (46.2%), immunosuppression de-escalation in 4 of 13 episodes (30.8%), non-immunosuppressive clinical actions in 2 of 13 episodes (15.4%) and maintenance of treatment in 1 of 13 episodes (7.7%).

## 4. Discussion

In this real-world single-centre cohort of patients with lupus nephritis, repeat kidney biopsy showed relevant diagnostic yield, characterized by frequent histological transitions and by the identification of a subgroup of patients in whom repeat biopsy did not support active lupus nephritis [26,34,35,36,37,38]. These findings suggest that repeat kidney biopsy can provide clinically meaningful information beyond conventional clinical and laboratory assessment, particularly in scenarios of diagnostic or therapeutic uncertainty [3,9,26,27,28,29]. In addition, sequential analysis of consecutive biopsies showed that part of the diagnostic yield of repeat biopsy emerges during longitudinal histological follow-up and may not be captured by analyses limited to the comparison between the index and the last biopsy [39,40,41,42,43,44].

The clinical relevance of these findings is supported by the well-recognized discordance between clinical biomarkers and renal histology in lupus nephritis [3,9,26,27,28,29]. Proteinuria, kidney function and immunological markers may not reliably distinguish active inflammatory lesions, incident membranous disease, chronic irreversible damage or alternative diagnoses [3,9,26,27,28,29]. In this context, repeat biopsy may function as a decision-support tool, helping clinicians to individualize treatment escalation, maintenance or de-escalation according to the underlying histological substrate [26,28,30,31,33,44].

Our findings are consistent with previous studies describing clinically relevant clinicopathological discordance in lupus nephritis and frequent histological changes in repeat biopsies [27,28,29,30,31,32,33,34,35,36,37,38]. In particular, several studies have shown that clinical remission does not necessarily imply the absence of residual histological activity and that persistent activity may be associated with renal relapse and worse long-term outcomes [27,28,29,30,31,32,33,39,40,41,42,43,44]. Similarly, studies focused on repeat biopsies performed during suspected flares or according to clinical indication have reported frequent class transitions and an added value of a second biopsy in characterizing the renal episode [34,35,36,37,38]. Large contemporary SLE cohorts, including Kosałka-Węgiel’s cohort comparing patients with and without LN, provide useful epidemiological context; however, they do not specifically address the diagnostic yield of repeat kidney biopsy [4,5,6,46].

In this context, our data reinforce the concept that the utility of repeat biopsy should not be reduced to class switching alone [3,8,9,26]. Rather, repeat kidney biopsy should be considered a tool to resolve discrepancies between clinical findings, serology and histology [3,9,26,27,28,29]. Prospective studies such as REBIOLUP (NCT04449991), which evaluates the utility of protocol repeat kidney biopsy compared with conventional clinical follow-up in patients with de novo lupus nephritis, will be relevant to define the role of scheduled repeat biopsy more precisely. In contrast, our cohort reflects routine clinical practice, where repeat biopsy was performed in heterogeneous scenarios of diagnostic or therapeutic uncertainty [26,34,35,36,37,38].

One clinically relevant finding in our cohort was the detection of incident membranous component during follow-up [7,37,38]. Sequential analysis identified class V lesions in more patients than would have been detected by comparing only the index and the last biopsy, suggesting that first-to-last biopsy analyses may underestimate intermediate histological changes with therapeutic implications [39,40,43,44]. This is particularly relevant because persistent or recurrent proteinuria may reflect proliferative activity, membranous lupus nephritis or chronic structural damage, each of which may lead to different therapeutic decisions [1,3,7,8,9,14,24,26]. In our cohort, the detection of class V lesions in repeat biopsy preceded the initiation of calcineurin inhibitor therapy in two patients, supporting the potential actionability of this finding [1,14,24].

Another relevant observation was the identification of diagnoses not compatible with active lupus nephritis in three patients [26,34,35,36]. This finding has a different clinical meaning from histological reclassification within the ISN/RPS system, because it may shift clinical reasoning away from treatable lupus activity and toward established damage or alternative diagnoses [3,7,8,9,26]. In such cases, repeat biopsy may help to avoid an unnecessary escalation of immunosuppression when histology does not support active lupus nephritis [26,28,30,31,33,44].

It is also noteworthy that repeat biopsy provided diagnostic information in scenarios other than overt clinical flare, including patients evaluated in the context of planned reduction or withdrawal of immunosuppression [28,29,30,31,33,44]. This observation is consistent with studies exploring the role of repeat biopsy to guide maintenance or withdrawal strategies, which have shown that histological activity may persist despite apparent clinical remission and may be associated with subsequent relapse [28,29,30,31,33,44]. Although our results do not allow firm prognostic conclusions, they suggest that repeat biopsy may not only be informative during clinical worsening or refractory disease, but also in selected patients in whom therapeutic de-escalation is being considered [28,30,31,33,44].

Other histopathological features beyond the ISN/RPS class may also provide clinically relevant information in lupus nephritis. In particular, tubulointerstitial inflammation, interstitial fibrosis and inflammatory cell composition have been associated with renal prognosis and may complement glomerular class-based assessment. Interstitial mast cells have been proposed as potential contributors to renal inflammation and fibrosis; previous studies have reported differences in tryptase-positive and chymase-positive mast cell counts across lupus nephritis classes and correlations with tubulointerstitial fibrosis and renal function parameters [47,48]. Moreover, interstitial tryptase-positive cells have been proven to be more common in class V LN when compared to primary membranous nephropathy, suggesting that they may have a key role in renal disease in SLE patients [49]. In the present study, mast cell counts and other immunohistochemical markers of interstitial inflammation were not systematically assessed in the original pathology reports, and no centralized retrospective pathological review was performed.

The main strength of this study is that it reflects real-world clinical practice, with heterogeneous indications for repeat biopsy that are close to the scenarios in which diagnostic or therapeutic uncertainty usually arises [26,34,35,36,37,38]. In addition, the analysis was not limited to the comparison between index and last biopsy but incorporated the sequential dynamics between consecutive biopsies [39,40,41,42,43,44]. This approach allowed us to capture intermediate findings that might be missed in a binary first-to-last analysis [39,40,43,44]. The study also emphasizes clinically meaningful dimensions of diagnostic yield, including incident membranous component and diagnoses not compatible with active lupus nephritis [26,34,35,36,37,38].

This study has several limitations. First, it was retrospective, single-centre and included a small number of patients, which limits the precision of the estimates and the generalizability of the findings. Second, indications for repeat biopsy were not protocolized, introducing an inherent risk of selection bias. Third, histological activity and chronicity indices were not homogeneously available across all biopsies, and no centralized retrospective pathological review was performed. This prevented the quantitative assessment of changes in activity and chronic damage and limits direct comparison with studies evaluating the prognostic value of residual activity or increasing chronicity on repeat biopsy [29,39,40,41,42,43,44]. Fourth, extra-renal manifestations were not systematically available and were therefore not included in the analysis [2,6,45]. Fifth, because of the retrospective design, repeat kidney biopsy could not be consistently matched with all clinical, urinary and serological parameters considered markers of lupus nephritis activity, including SLEDAI, renal SLEDAI, standardized active urinary sediment definitions, 24 h proteinuria, anti-dsDNA antibody titres and complement levels. This limited our ability to assess associations between activity parameters and repeat-biopsy histology. Finally, although diagnostic yield was assessed using clinically meaningful categories, these definitions should be considered exploratory and not equivalent to externally validated decision tools [44].

Because of the retrospective design and limited sample size, our findings should be interpreted as hypothesis-generating. The results do not establish a universal indication for repeat kidney biopsy in all patients with lupus nephritis but rather support its potential value in selected scenarios where clinical, serological and histological uncertainty may affect therapeutic decision-making [1,3,8,9,26,28,30,31,32,33,44].

Future studies should focus on larger multicenter cohorts with homogeneous definitions of repeat biopsy indications, systematic assessment of histological activity and chronicity indices, and specific analyses of clinically relevant scenarios such as immunosuppression withdrawal, persistent proteinuria with inactive serology, suspected transition toward membranous lesions, or discordance between clinical response and histological activity [3,8,29,39,40,44]. The integration of longitudinal histological information with clinical and serological biomarkers may help to define more precise strategies for monitoring, treatment escalation and therapeutic de-escalation in lupus nephritis [1,2,3,8,29,30,31,39,40,44].

## 5. Conclusions

In this real-world single-centre cohort, repeat kidney biopsy in lupus nephritis provided clinically relevant diagnostic information, not only by identifying ISN/RPS class changes among biopsies still classified as lupus nephritis, but also by detecting incident membranous component and diagnoses not compatible with active lupus nephritis. Sequential analysis of consecutive biopsies identified intermediate findings that could be missed by analyses restricted to the index and last biopsy. Although these results should be interpreted in the context of a retrospective study with small sample size and heterogeneous indications, they support the role of repeat kidney biopsy as a tool to resolve clinicopathological uncertainty and guide individualized therapeutic decisions in selected patients.

## Figures and Tables

**Table 1:** Baseline characteristics of the cohort at index biopsy.

Variable	Value
Women, *n* (%)	16 (84.2)
Age at SLE diagnosis, years	33.0 (21.5–40.0)
Age at LN diagnosis, years	35.0 (24.0–41.0)
Follow-up until last biopsy, months	60.2 (50.4–114.5)
Biopsies per patient, median (range)	2 (2–5)
Total biopsies, *n*	45
Index class I, *n* (%)	3 (15.8)
Index class III, *n* (%)	8 (42.1)
Index class IV, *n* (%)	6 (31.6)
Index class III + V, *n* (%)	2 (10.5)
Baseline creatinine, mg/dL	0.81 (0.62–1.18)
Baseline eGFR, mL/min/1.73 m^2^	90 (53–90)
Baseline proteinuria, mg/g	912 (683.4–2000.5)
Baseline hematuria	51.0 (11.9–90.2)
Baseline leukocyturia	27.3 (13.2–60.0)
Baseline anti-dsDNA, IU/mL	116.1 (45.0–666.9)
Baseline C3, mg/dL	78.0 (54.1–92.3)
Baseline C4, mg/dL	11.0 (8.0–18.0)

**Table 2:** Clinical indications for repeat kidney biopsy: episode-level analysis.

Indication	Episodes, *n*	%
Serological and/or proteinuric worsening	13	50.0
Reduction/withdrawal of immunosuppression	7	26.9
Refractory disease	3	11.5
Inclusion in clinical trial	3	11.5

**Table 3:** Diagnostic yield at patient level: comparison between index and last biopsy.

Finding	*n*/*N*	%
Histological or diagnostic class change	14/19	73.7
Lupus nephritis class change	11/19	57.9
New membranous component in the last biopsy	2/19	10.5
New membranous component at any time during follow-up	4/19	21.1
Diagnosis not compatible with active LN in the last biopsy	3/19	15.8

**Table 4:** Diagnostic yield at repeat biopsy according to clinical indication.

Indication	Episodes	Lupus Nephritis Class Change *n* (%)	New Membranous Component *n* (%)	Not Active LN, *n* (%)	Any Relevant Finding *n* (%)
Serological and/or proteinuric worsening	13	7 (53.8)	2 (15.4)	2 (15.4)	9 (69.2)
Reduction/withdrawal of immunosuppression	7	6 (85.7)	1 (14.3)	1 (14.3)	7 (100)
Refractory disease	3	2 (66.7)	1 (33.3)	0	2 (66.7)
Inclusion in clinical trial	3	2 (66.7)	0	0	2 (66.7)
Total	26	17 (65.4)	4 (15.4)	3 (11.5)	20 (76.9)

A clinically relevant diagnostic finding was defined as the presence of at least one of the following elements in a transition between consecutive biopsies: ISN/RPS class change among biopsies classified as lupus nephritis, incident membranous component not present in the immediately preceding biopsy or a diagnosis not compatible with active lupus nephritis. LN: lupus nephritis.

**Table 5:** Therapeutic consequences after repeat kidney biopsy.

Therapeutic Consequence After Repeat Biopsy	Repeat Biopsy, *n*/*N* (%)	Details
Immunosuppression escalation/intensification	10/26 (38.5)	Cyclophosphamide initiation, methylprednisolone pulses and corticosteroid intensification, mycophenolic acid initiation/increase, CNI initiation, rituximab-based escalation or combined escalation strategies
Immunosuppression de-escalation/withdrawal	6/26 (23.1)	Mycophenolic acid reduction, corticosteroid withdrawal, tacrolimus withdrawal or broader immunosuppression reduction
Maintenance/no treatment reduction	6/26 (23.1)	No immediate treatment reduction after repeat biopsy
Clinical trial inclusion	2/26 (7.7)	Repeat biopsy followed by inclusion in a clinical trial
Non-immunosuppressive clinical action	2/26 (7.7)	ACE inhibitor initiation or kidney replacement therapy initiation

## Data Availability

The data underlying this study are not publicly available due to privacy and ethical restrictions related to patient-level clinical information. Anonymized data may be made available from the corresponding author upon reasonable request and subject to approval by the relevant ethics committee.

## Abbreviations

The following abbreviations are used in this manuscript

Anti-dsDNA	Anti-double-stranded DNA antibody
eGFR	Estimated Glomerular Filtration Rate
ISN/RPS	International Society of Nephrology/Renal Pathology Society
LN	Lupus nephritis
SLE	Systemic lupus erythematosus
UPCR	Urine protein-to-creatinine ratio
CNI	Calcineurin inhibitor
